# Construction of Pool-Boiling Curves for a CNT Buckypaper Layer on Copper Based on Reference Cu–LN_2_ Curves

**DOI:** 10.3390/ma19214743

**Published:** 2026-11-08

**Authors:** Zbigniew A. Łagodowski, Łukasz Stępień, Grzegorz Komarzyniec

**Affiliations:** 1 Department of Mathematics, Lublin University of Technology, 38A Nadbystrzycka Street, 20-618 Lublin, Poland; z.lagodowski@pollub.pl; 2 Department of Electrical Engineering and Superconductivity Technologies, Lublin University of Technology, 38A Nadbystrzycka Street, 20-618 Lublin, Poland; g.komarzyniec@pollub.pl

**Keywords:** cryogenic pool boiling, complete boiling curve, liquid nitrogen, copper surface, carbon nanotube buckypaper, electrothermal modeling

## Abstract

This study develops an operator-based method for constructing scenario-based boiling curves for a carbon nanotube buckypaper (BP) layer on copper in liquid nitrogen, using a reference Cu–LN_2_ characteristic. The reference curve is constructed using established cryogenic pool-boiling correlations, complemented by appropriate connections between the boiling regimes. The proposed operator transforms the reference characteristic to represent potential changes in boiling behavior associated with the BP layer. Two scenarios, moderately optimistic and optimistic, are considered for three surface orientations: 0°, 90°, and 180°. The resulting complete and continuous boiling curves can be used as boundary conditions in thermal and electrothermal models. The transformation parameters are scenario-based and require experimental calibration for the BP–Cu–LN_2_ system.

## 1. Introduction

Coupled electromagnetic–thermal analyses and studies of transient states in tapes and devices employing high-temperature superconductors (HTSs) indicate the need for a simultaneous description of heat generation and heat removal to liquid nitrogen (LN_2_); see, e.g., [1,2,3,4]. For the reference Cu–LN_2_ interface, the cooling description requires the characteristic $q_{\mathrm{Cu}}^{\prime \prime }\left(\Delta T;\theta \right)$, where Δ*T* is the surface superheat and *θ* denotes the orientation angle of the heating surface. This characteristic can be used as a boundary condition in electrothermal models. The direct motivation for the present work is the need to construct a reference cooling characteristic for the Cu–LN_2_ interface and to assess the possibility of improving cooling by a carbon nanotube (CNT) buckypaper layer (hereafter: BP) deposited on Cu.

**Figure 1:**
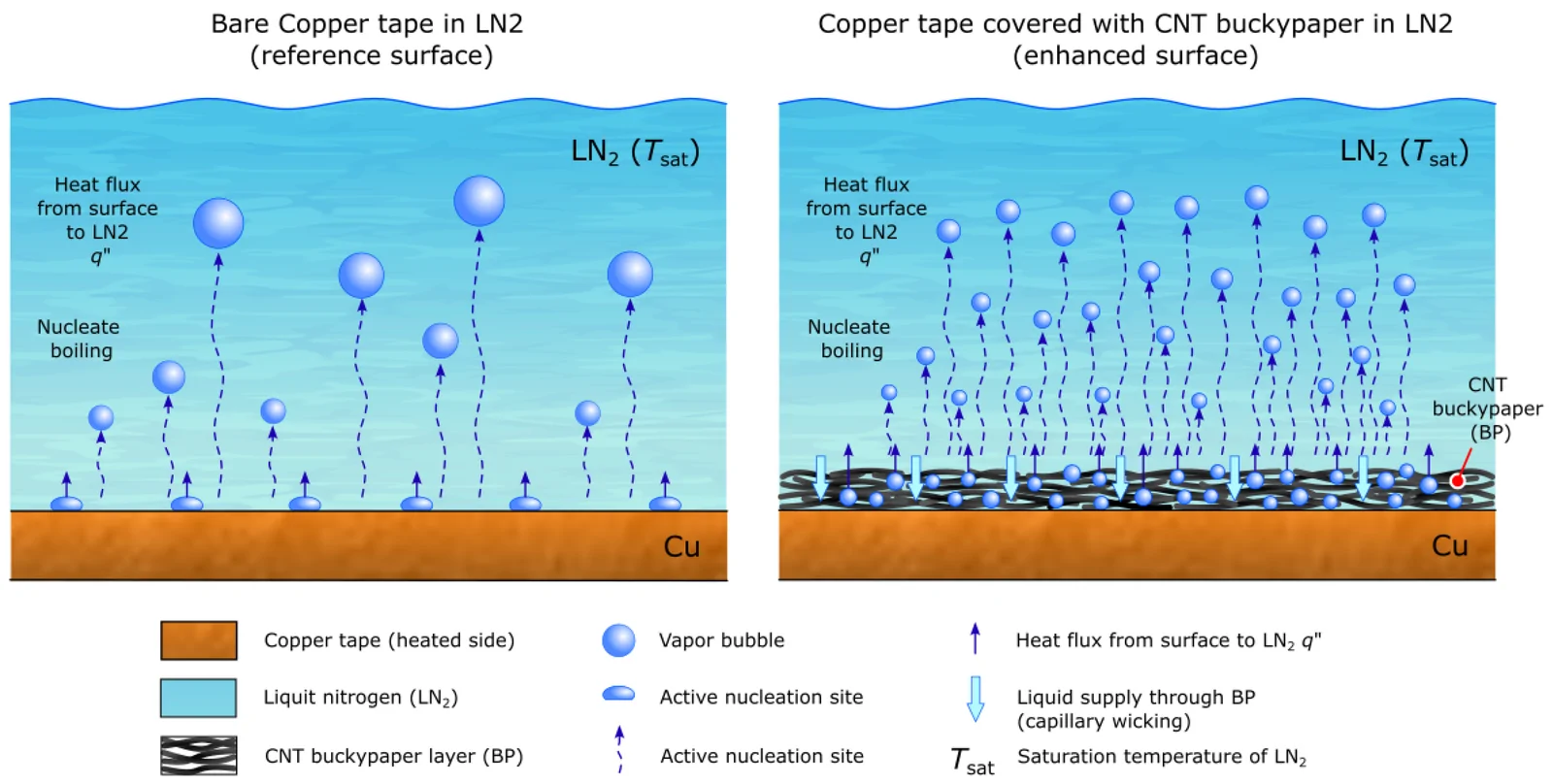
Conceptual comparison of nucleate boiling on a bare Cu surface (**left**) and on a Cu surface covered with CNT buckypaper (**right**).

A CNT buckypaper layer is considered for two complementary reasons. Its porous structure may increase the number of active nucleation sites, improve wettability, and facilitate capillary liquid supply to the heated surface, leading to a reduction in the superheat required to dissipate a given heat flux and to an increase in critical heat flux (CHF) [5,6]. These mechanisms are illustrated schematically in Figure 1.

At the same time, individual carbon nanotubes are characterized by very high axial thermal conductivity. Long phonon mean free paths may lead, depending on nanotube length and transport conditions, to partly ballistic behavior, whereas, for sufficiently long CNTs, superdiffusive transport has also been predicted [7,8,9,10,11]. Results obtained for individual nanotubes cannot be directly identified with the properties of macroscopic CNT buckypaper because its effective conductivity also depends on nanotube alignment, bundle continuity, and thermal contact resistances at their junctions [12,13]. Sufficiently densified BP layers may, however, retain high in-plane conductivity and act as heat-spreading elements [14].

In the system considered here, in-plane conduction in the BP may provide an additional path for lateral heat spreading along the tape. In the event of a local loss of the superconducting state, part of the heat generated in the resistive region may be distributed to a larger area in contact with LN_2_, which may reduce local temperature gradients and the maximum temperature of the hot region. This mechanism motivates further development of coupled electromagnetic–thermal models of HTS tapes and devices [1]. Studies of CNT applications in device-cooling technologies include thermal interfaces between a heat source and a heat sink [15] and anisotropic heat-spreading layers intended to reduce local hot spots in integrated circuits [16], as well as capillary wick structures in two-phase heat spreaders and loop heat pipes [17,18]. CNT sheets have also been investigated as passive heat-dissipating surfaces under natural-convection conditions [19]. In the present work, lateral heat spreading in the BP layer is not yet modeled quantitatively.

The presence of an additional layer does not, however, automatically lead to improved cooling because the BP layer and its contact with Cu introduce an additional thermal resistance

$$R_{\mathrm{add}}^{\prime \prime }=R_{\mathrm{Cu}-\mathrm{BP}}^{\prime \prime }+\frac{t_{\mathrm{BP}}}{k_{⊥,\mathrm{BP}}},$$

where $R_{\mathrm{Cu}-\mathrm{BP}}^{\prime \prime }$ is the area-specific thermal contact resistance of the Cu–BP interface, *t*_BP_ is the layer thickness, and *k*_⊥,BP_ is its effective thermal conductivity normal to the surface. For a prescribed heat flux and a fixed monotonic segment of the curve, the BP layer improves cooling of the Cu surface if

(1) $$\Delta T_{\mathrm{BP}}\left(q^{\prime \prime };\theta \right)+q^{\prime \prime }R_{\mathrm{add}}^{\prime \prime }<\Delta T_{\mathrm{Cu}}\left(q^{\prime \prime };\theta \right),$$

where Δ*T*_Cu_ and Δ*T*_BP_ denote the superheats required to dissipate the same heat flux through the Cu–LN_2_ and BP–LN_2_ interfaces, respectively, evaluated on corresponding monotonic segments of the two curves. Condition (1) applies to local, quasi-steady heat transport normal to the layer; lateral heat spreading in its plane constitutes an additional cooling-enhancement mechanism.

There are indications that the additional thermal resistance of the BP layer and the Cu–BP contact will not eliminate the benefits arising from improved heat transfer at the BP–LN_2_ interface. The thickness *t*_BP_ is a design parameter; therefore, the term

$$\frac{t_{\mathrm{BP}}}{k_{⊥,\mathrm{BP}}}$$

can be limited by using a sufficiently thin layer. Moreover, studies of CNT structures used as thermal interfaces with Cu indicate that appropriate mechanical pressure can increase the thermal contact conductance [15]. For densified and flattened CNT layers, increasing the real contact area also reduced the contact resistance [16]. Densification of nanotube networks additionally improves the continuity of heat-transport paths in CNT buckypaper layers [14]. These results do not directly concern a BP layer on Cu in liquid nitrogen, but they support the assumption that, with appropriately selected thickness and attachment to the substrate, the additional resistance $R_{\mathrm{add}}^{\prime \prime }$ need not be the dominant factor. At the same time, studies of CNT layers on metallic substrates indicate an increase in the heat transfer coefficient in the nucleate-boiling regime and an increase in CHF [5,6]. The first of these effects corresponds to a reduction in the superheat required to dissipate a given heat flux in this regime, whereas the second extends the range of heat fluxes attainable before the boiling crisis occurs. Taken together, these results provide evidence that the improvement in heat transfer at the BP–LN_2_ interface may exceed the additional temperature drop across the BP layer and its contact with Cu, so that condition (1) may be satisfied.

Final confirmation of this condition for the system considered requires determination of $R_{\mathrm{Cu}-\mathrm{BP}}^{\prime \prime }$, *k*_⊥,BP_, the thickness *t*_BP_, and the anisotropic properties of buckypaper at cryogenic temperatures.

A complete assessment of whether the BP layer improves cooling requires comparison of the heat-transfer characteristics at the Cu–LN_2_ and BP–LN_2_ interfaces. Under pool-boiling conditions, these characteristics can be written as

$$q^{\prime \prime }=q^{\prime \prime }\left(\Delta T\right),\;\Delta T=T_{s}-T_{\mathrm{sat}},$$

where *T*_*s*_ is the temperature of the surface in contact with the liquid. Rohsenow [20] developed a classical correlation for nucleate boiling, whereas Berenson [21] described heat transfer in the film-boiling regime. Correlations for individual regimes, however, do not automatically form a single continuous characteristic spanning the full range of superheat. Ahmad et al. [22] presented a method for generating a complete, continuous saturated pool-boiling curve for cryogenic fluids. In a subsequent study, Ahmad et al. [23] extended this method to include the effects of surface properties and boiling conditions, including roughness, material and surface size, orientation, pressure, and liquid subcooling. Bombardieri and Manfletti [24] investigated the influence of wall material on nucleate boiling of liquid nitrogen, whereas Cai, Mudawar, and Liu [25] presented a method for determining the surface temperature at the minimum heat flux point. These studies provide the basis for construction of the reference boiling curve for the Cu–LN_2_ interface.

For the purposes of the present work, the methodology of Ahmad et al. is used to prepare a specific reference representation of the Cu–LN_2_ characteristic for the adopted system parameters and surface orientation. The relations describing the individual regimes are written as explicit functions of superheat wherever this is possible and useful computationally. The resulting characteristic $q_{\mathrm{Cu}}^{\prime \prime }\left(\Delta T;\theta \right)$ then serves as the reference curve for the model-based transformation to the characteristic of the CNT buckypaper layer deposited on Cu.

The second interface considered is the BP–LN_2_ interface, formed by the CNT buckypaper layer deposited on the Cu surface and in contact with liquid nitrogen. No complete boiling curve for such a system was found in the literature. The available studies concern different nanotube structures, substrates, working fluids, and surface orientations. Ujereh, Fisher, and Mudawar [26] investigated the effect of CNT layers on boiling on flat and microstructured Cu and Si substrates, demonstrating a strong dependence of the results on nanotube density, distribution, and surface coverage. Dharmendra et al. [27] analyzed boiling on vertically aligned CNT layers deposited on a Cu substrate and demonstrated improved boiling performance relative to uncoated copper. McHale et al. [28] compared smooth and sintered Cu surfaces with and without CNT layers, indicating that the influence of nanotubes also depends on the substrate microstructure.

Mao, Hu, and Zhou [5] obtained, for a nanoporous multi-walled carbon nanotube (MWCNT) coating on Cu, an increase in critical heat flux and maximum heat transfer coefficient. Lee et al. [6] showed that multilayer polyethylenimine (PEI)–MWCNT coatings on a type 316 stainless steel (SS316) substrate can increase both the heat transfer coefficient and CHF, although the configurations most favorable for the two quantities need not be identical. Ho, Leong, and Yang [29] investigated CNT layers at different surface orientations, confirming that orientation may affect the observed boiling characteristic. Zhou et al. [30] used commercial CNT buckypaper to enhance boiling of FC-72, a perfluorocarbon dielectric liquid. This work is particularly relevant because of the form of the CNT layer, but the working fluid used does not allow the reported values to be transferred directly to LN_2_ boiling.

Further evidence is provided by studies of porous surfaces conducted directly in liquid nitrogen. Surtaev et al. [31] demonstrated the effect of the thickness and morphology of capillary-porous coatings on heat-transfer intensity, hysteresis, and the development of the boiling crisis. Kuznetsov and Pavlenko [32] observed, for microstructured porous coatings on copper heaters, an increase in CHF, enhancement in heat transfer, and a change in the slope of the curve in the pre-critical range. These studies do not concern CNT buckypaper and therefore serve primarily for a qualitative assessment of possible changes in the transition-boiling branch and the conditions for transition to film boiling. Additional quantitative evidence concerning possible changes in the coordinates of the minimum heat flux (MHF) point is provided by results reported by Hu et al. and Sathyamurthi et al. [33,34]; their relevance to the selection of the ranges of the MHF-point scaling factors *ξ*_1_ and *ξ*_2_, applied to the superheat and heat-flux coordinates, respectively, is discussed later in the paper.

When selecting the supporting evidence, priority is given to results concerning CNT layers or buckypaper deposited on Cu. As a second priority, results for CNT layers on other metallic substrates are used, whereas studies of other porous surfaces in LN_2_ are used mainly to assess the direction of possible changes. This distinction is necessary because boiling properties depend not only on the presence of nanotubes but also on the layer architecture, its contact with the substrate, wettability, porosity, capillary transport, working fluid, and surface orientation.

Although the available studies provide evidence of boiling enhancement on CNT-modified and porous surfaces, they do not provide a complete experimental boiling characteristic for the BP–Cu–LN_2_ system considered here. Their results cannot be transferred directly because of differences in working fluids, substrates, and layer structures. This creates a need for a method that combines a complete reference characteristic with incomplete experimental evidence to construct continuous, scenario-based boiling curves for use in thermal and electrothermal models. The main objective of this work is therefore to develop an operator-based method for transforming a reference Cu–LN_2_ boiling curve into a complete BP–LN_2_ interface characteristic. As a prerequisite, the reference curve is constructed using the methodology of Ahmad et al. [22] and its updated parametric correlations [23], specialized to the system parameters and surface orientations considered. The proposed operator modifies the characteristic points while preserving the normalized shapes of the transformed branches and continuity at their junctions. Its parameters define literature-informed scenarios and can subsequently be calibrated against experimental data for the target system. The resulting interface characteristics are intended as boundary conditions for thermal and electrothermal analyses, including models of superconducting tapes and devices cooled with liquid nitrogen.

## 2. Reference Cu–LN_2_ Pool-Boiling Curve

The heat-transfer process in saturated liquid nitrogen is briefly outlined below. At low surface superheat, heat is transferred to liquid nitrogen by natural convection (NC). At the onset of nucleate boiling (ONB), vapor bubbles begin to form at the heated surface, initiating nucleate boiling (NB). As the CHF point is approached, bubble coalescence increasingly hinders liquid replenishment at the surface. In transition boiling (TB), between CHF and MHF, unstable vapor coverage limits liquid–surface contact, and the heat flux decreases with increasing surface superheat. At higher superheats, film boiling (FB) is characterized by a continuous vapor layer separating the heated surface from the liquid.

### Methodology for Determining the Boiling Characteristic $q_{\mathrm{Cu}}^{\prime \prime }\left(\Delta T;\theta \right)$

Unless stated otherwise, all dimensional quantities in the correlations below are expressed in SI units. In particular, heat flux is given in W/m^2^ and heat transfer coefficients in $W/(m^{2}\;K)$.

Ahmad, Foster, Kim, Meyer, Hartwig, and Mudawar [22] (pp. 4–7) presented a method for generating a complete boiling curve by combining relations describing the successive regimes and the ONB, CHF, and MHF points. In the present work, this methodology is applied to determine the boiling characteristic for the Cu–LN_2_ system for the adopted surface parameters, saturated pool-boiling conditions, and a fixed orientation *θ*. The complete reference boiling characteristic is expressed in the following general, piecewise form:

(2) $$q_{\mathrm{Cu}}^{\prime \prime }\left(\Delta T;\theta \right)=\left\{\begin{array}{cc} q_{\mathrm{NC}}^{\prime \prime }\left(\Delta T;\theta \right),\; & \Delta T_{L,4}\leq \Delta T\leq \Delta T_{\mathrm{ONB}}\left(\theta \right),\\ q_{\mathrm{NB}}^{\prime \prime }\left(\Delta T;\theta \right),\; & \Delta T_{\mathrm{ONB}}\left(\theta \right)<\Delta T\leq \Delta T_{\mathrm{CHF}}\left(\theta \right),\\ q_{\mathrm{TB}}^{\prime \prime }\left(\Delta T;\theta \right),\; & \Delta T_{\mathrm{CHF}}\left(\theta \right)<\Delta T\leq \Delta T_{\mathrm{MHF}},\\ q_{\mathrm{blend}}^{\prime \prime }\left(\Delta T;\theta \right),\; & \Delta T_{\mathrm{MHF}}<\Delta T\leq \Delta T_{\mathrm{FB},1.5}\left(\theta \right),\\ q_{\mathrm{FB}}^{\prime \prime }\left(\Delta T;\theta \right),\; & \Delta T>\Delta T_{\mathrm{FB},1.5}\left(\theta \right). \end{array}\right.$$

In this section, we present the explicit formulas for $q_{\mathrm{NC}}^{\prime \prime }$, $q_{\mathrm{NB}}^{\prime \prime }$, $q_{\mathrm{TB}}^{\prime \prime }$, $q_{\mathrm{blend}}^{\prime \prime }$, and $q_{\mathrm{FB}}^{\prime \prime }$, and explain how the characteristic points defining the corresponding intervals in Equation (2) are determined. For the NB branch, the correlation for the heat transfer coefficient given in [23] (Equation (9)) is combined with $q^{\prime \prime }=h\Delta T$, and the resulting equation is solved for $q^{\prime \prime }$. The TB relation [23] (Equations (22) and (23)) and the MHF–FB blending relation [22] (Equations (17)–(18)) express superheat as a function of heat flux and are therefore inverted to obtain explicit expressions for $q^{\prime \prime }\left(\Delta T\right)$. The NC and FB branches are obtained by determining *h*_NC_(Δ*T*; *θ*) and *h*_FB_(Δ*T*; *θ*) from the corresponding correlations [23] (Section 2.2, Equations (1) and (2), p. 4) and [23] (Equation (31), p. 17), respectively, and then using the general relation to obtain $q_{\mathrm{NC}}^{\prime \prime }\left(\Delta T;\theta \right)$ and $q_{\mathrm{FB}}^{\prime \prime }\left(\Delta T;\theta \right)$. The ONB point and the junction point between the blending curve and the FB branch are determined by solving the corresponding equations numerically. The coordinates of the CHF and MHF points are obtained from the Ahmad correlations after substituting the parameters of the system considered.

The orientation angle *θ* is measured from the horizontal upward-facing surface:

$\begin{array}{cccc} \theta & =0^{\circ }, & \; & \mathrm{horizontal}\;\mathrm{upward}-\mathrm{facing}\;\mathrm{surface},\\ \theta & =90^{\circ }, & & \mathrm{vertical}\;\mathrm{surface},\\ \theta & =180^{\circ }, & & \mathrm{horizontal}\;\mathrm{downward}-\mathrm{facing}\;\mathrm{surface}. \end{array}$

The dependence on *θ* follows the source correlation used for each boiling regime; any restrictions on the orientation range are specified together with the corresponding correlation.

In all correlations involving *θ*, its numerical value is expressed in degrees, including in the trigonometric functions.

Natural convection.

Ahmad et al. [23] (Section 2.2, Equations (1) and (2), p. 4) provide an empirical relation that allows the heat transfer coefficient *h*_NC_ to be determined:

$$Nu_{\mathrm{NC}}=\frac{h_{\mathrm{NC}}L_{c}}{k_{l}}=C\;Ra_{L}^{\;n},$$

where *Nu*_NC_ is the Nusselt number, *L*_*c*_ is the minimum characteristic dimension of the heating surface, i.e., the smaller of its length and width [m], *k*_*l*_ is the thermal conductivity of the single-phase liquid, and *C* and *n* are empirical constants depending on surface orientation and the Rayleigh-number (*Ra*_*L*_) range; see Table 1. The Rayleigh number is given by Ahmad et al. [23] (Equation (2), p. 4):

(3) $$Ra_{L}\left(\Delta T\right)=\frac{L_{c}^{3}\;\rho_{l}\left[\rho_{l}-\rho_{l,w}\left(\Delta T\right)\right]g}{\mu_{l}^{2}}Pr_{l}.$$

Here, *g* is the gravitational acceleration and *ρ*_*l*_ = *ρ*_*l*_(*T*_*f*_) denotes the liquid density at the bulk-liquid temperature *T*_*f*_ = *T*_sat_, whereas *ρ*_*l*,*w*_(Δ*T*) = *ρ*_*l*_(*T*_sat_ + Δ*T*) denotes the liquid density evaluated at the surface temperature. The factor *Pr*_*l*_ is the Prandtl number of the single-phase liquid:

$$Pr_{l}=\frac{\mu_{l}c_{p,l}}{k_{l}}.$$

The quantities *μ*_*l*_, *c*_*p*,*l*_, and *k*_*l*_ are, respectively, the dynamic viscosity, specific heat, and thermal conductivity of the (single-phase) liquid.

The superheat values corresponding to the Rayleigh-number range boundaries are determined from the equation

$$Ra_{L}\;\left(\Delta T_{L,m}\right)=10^{m},\;m\in \left\{4,7,9,11,13\right\}.$$

For the NC correlation, Ahmad et al. provide the coefficients *C* and *n* for *θ* = 0°, 90°, and 180° [23] (Table 1, p. 4).

Within the natural-convection regime considered, *Ra*_*L*_(Δ*T*) is an increasing function; therefore, successive limiting values of the Rayleigh number can be uniquely assigned to the corresponding superheat values. After applying the coefficients from Table 1, the natural-convection correlation takes the form

(4) $$q_{\mathrm{NC}}^{\prime \prime }\left(\Delta T;\theta \right)=\frac{k_{l}}{L_{c}}\;\Delta T\left\{\begin{array}{cc} 0.54\;{\left[Ra_{L}\left(\Delta T\right)\right]}^{1/4},\; & \theta =0^{\circ },\;\Delta T_{L,4}\leq \Delta T\leq \Delta T_{L,7},\\ 0.15\;{\left[Ra_{L}\left(\Delta T\right)\right]}^{1/3},\; & \theta =0^{\circ },\;\Delta T_{L,7}<\Delta T\leq \Delta T_{L,11},\\ 0.59\;{\left[Ra_{L}\left(\Delta T\right)\right]}^{1/4},\; & \theta =90^{\circ },\;\Delta T_{L,4}\leq \Delta T\leq \Delta T_{L,9},\\ 0.10\;{\left[Ra_{L}\left(\Delta T\right)\right]}^{1/3},\; & \theta =90^{\circ },\;\Delta T_{L,9}<\Delta T\leq \Delta T_{L,13},\\ 0.52\;{\left[Ra_{L}\left(\Delta T\right)\right]}^{1/5},\; & \theta =180^{\circ },\;\Delta T_{L,4}\leq \Delta T\leq \Delta T_{L,9}. \end{array}\right.$$

The applicability ranges of the individual cases also include additional conditions concerning the Prandtl number, given in Table 1. For intermediate values of *θ*, the work of Ahmad et al. does not provide an interpolation of the coefficients *C* and *n*.

Equation (4) defines the natural-convection correlation for the orientations included in Table 1. In the complete boiling curve, for a fixed value of *θ*, the NC branch is the restriction of the corresponding case of this correlation to the interval

$$\Delta T_{L,4}\leq \Delta T\leq \Delta T_{\mathrm{ONB}}\left(\theta \right).$$

For *θ* = 0° or 90°, if the ONB point is reached before the threshold separating the two correlation ranges is exceeded, the NC branch contains only the first of the cases corresponding to the given orientation in Equation (4). For *θ* = 180°, the source correlation covers a single Rayleigh-number range. The formal left endpoint of the source-correlation range is Δ*T*_*L*,4_; its extension to Δ*T* = 0 will be specified at the numerical implementation stage.

ONB point.

The onset-of-nucleate-boiling (ONB) point,

$$\left(\Delta T_{\mathrm{ONB}}\left(\theta \right),q_{\mathrm{ONB}}^{\prime \prime }\left(\theta \right)\right),$$

is determined by the intersection of the NC and NB branches. Its coordinates therefore satisfy

$$q_{\mathrm{NC}}^{\prime \prime }\left(\Delta T_{\mathrm{ONB}}\left(\theta \right);\theta \right)=q_{\mathrm{NB}}^{\prime \prime }\left(\Delta T_{\mathrm{ONB}}\left(\theta \right);\theta \right)=q_{\mathrm{ONB}}^{\prime \prime }\left(\theta \right).$$

The above equation for Δ*T*_ONB_(*θ*) is solved numerically.

Nucleate boiling.

Equation (9) of Ahmad et al. [23] (p. 7) includes the liquid subcooling Δ*T*_sub_ and the heating-surface conductivity ratio *k*_*w*_/*k*_Cu_. For the saturated liquid-nitrogen bath and Cu surface considered here,

$$\Delta T_{\mathrm{sub}}=T_{\mathrm{sat}}-T_{f}=0,\;\frac{k_{w}}{k_{\mathrm{Cu}}}=1.$$

Hence, that equation reduces to

(5) $$\begin{array}{cc} h_{\mathrm{NB}}= & 13.3\;{\left(q^{\prime \prime }\right)}^{0.665}{\left(1+0.52p^{*}\right)}^{4.7}\left(1+68e^{20(p^{*}-1.1)}\right)Pr_{f}^{-1.09}\\ \; & \times \left[1+\frac{1.5}{1+e^{-30(R_{a}-0.32)}}\right]\left(1+0.04\sin\theta \right). \end{array}$$

Here, p* = *p*/*p*_*c*_ is the reduced pressure; it is the quotient of the system pressure *p* and the critical pressure *p*_*c*_ of nitrogen. Furthermore,

$$Pr_{f}=\frac{\mu_{f}c_{p,f}}{k_{f}}$$

is the Prandtl number of the saturated liquid; *μ*_*f*_, *c*_*p*,*f*_, and *k*_*f*_ denote, respectively, its dynamic viscosity, specific heat, and thermal conductivity. In the present calculation, Prandtl numbers in (3) and in (5) are evaluated using the same liquid properties, so *Pr*_*f*_ = *Pr*_*l*_. The symbol *R*_*a*_ denotes the arithmetic mean deviation of the surface roughness profile, expressed in μm. The multiplier

1+0.04sinθ

describes the effect of surface orientation on the heat transfer coefficient in the nucleate-boiling regime. This multiplier was developed for 0° ≤ *θ* ≤ 90° [23] (Equation (7) and text below, p. 6). To determine the complete characteristic for *θ* = 180°, we extrapolate its functional form to this orientation, treating this extension as a modeling assumption.

Substituting *h*_NB_ from Equation (5) into $q_{\mathrm{NB}}^{\prime \prime }=h_{\mathrm{NB}}\Delta T$ and solving for $q_{\mathrm{NB}}^{\prime \prime }$, we obtain

$$q_{\mathrm{NB}}^{\prime \prime }\left(\Delta T;\theta \right)={\left(K_{\mathrm{NB}}\left(\theta \right)\Delta T\right)}^{1/0.335},$$

where

$$\begin{array}{cc} K_{\mathrm{NB}}\left(\theta \right)= & 13.3\;{\left(1+0.52p^{*}\right)}^{4.7}\left(1+68e^{20(p^{*}-1.1)}\right)Pr_{f}^{-1.09}\\ \; & \times \left[1+\frac{1.5}{1+e^{-30(R_{a}-0.32)}}\right]\left(1+0.04\sin\theta \right). \end{array}$$

CHF point.

The critical-heat-flux (CHF) point $\left(\Delta T_{\mathrm{CHF}}\left(\theta \right),q_{\mathrm{CHF}}^{\prime \prime }\left(\theta \right)\right)$ is established as follows. The critical heat flux is given by

$$\begin{array}{cc} q_{\mathrm{CHF}}^{\prime \prime }\left(\theta \right)= & \left[0.05{\left(p^{*}\right)}^{0.2}-0.104{\left(p^{*}\right)}^{12}+0.12\right]\left[1-0.004\;p^{*}\theta \right]{\left|\cos\;\left(\frac{88}{180}\theta \right)\right|}^{0.364}\\ \; & \times \left[\rho_{g}h_{fg}{\left(\frac{\sigma g(\rho_{f}-\rho_{g})}{\rho_{g}^{2}}\right)}^{1/4}\right]\\ \; & \times \left[e^{-1.7{\left(L_{c}/\left(3\lambda_{d}\right)\right)}^{-0.4}}+1\right]\left[0.49{\left(\frac{k_{\mathrm{Cu}}}{k_{f}}\right)}^{0.065}\right]. \end{array}$$

This is Equation (11) of Ahmad et al. [23] (p. 9) specialized to saturated pool boiling on a Cu surface under terrestrial gravity. The symbol *σ* denotes the liquid surface tension, while *λ*_*d*_ is the most dangerous Taylor wavelength:

$$\lambda_{d}=2\pi \sqrt{3}{\left[\frac{\sigma }{g(\rho_{f}-\rho_{g})}\right]}^{1/2},$$

see [23] (Equation (12), p. 10). Furthermore, *ρ*_*f*_ and *ρ*_*g*_ are the densities of the saturated liquid and vapor, respectively, *h*_*fg*_ is the latent heat of vaporization, and *k*_Cu_ is the thermal conductivity of copper. The corresponding superheat follows by evaluating the NB relation at $q_{\mathrm{CHF}}^{\prime \prime }\left(\theta \right)$. Thus,

$$\Delta T_{\mathrm{CHF}}\left(\theta \right)=\frac{{\left(q_{\mathrm{CHF}}^{\prime \prime }\left(\theta \right)\right)}^{0.335}}{K_{\mathrm{NB}}\left(\theta \right)}.$$

MHF point.

The coordinates of the minimum-heat-flux (MHF) point, $\left(\Delta T_{\mathrm{MHF}},q_{\mathrm{MHF}}^{\prime \prime }\left(\theta \right)\right),$ are obtained as follows. After substituting *k*_*w*_ = *k*_Cu_ into [23] (Equation (16), pp. 10–11), the formula for surface temperature at the MHF point reduces to

(6) $$T_{\mathrm{MHF}}=\left[T_{\mathrm{sat}}+1.6\left(T_{c}-T_{\mathrm{sat}}\right)\left(0.15+1.74{\left(p^{*}\right)}^{1/2}\right)\right]{\left(\frac{k_{\mathrm{Cu}}}{1+k_{\mathrm{Cu}}}\right)}^{0.07}.$$

The corresponding superheat is

$$\Delta T_{\mathrm{MHF}}=T_{\mathrm{MHF}}-T_{\mathrm{sat}}.$$

In Equation (6), *T*_*c*_ denotes the critical temperature of nitrogen, whereas the value of *k*_Cu_ must be entered in W/(mK). Equation (21) in [23] (p. 12) gives

$$\begin{array}{cc} q_{\mathrm{MHF}}^{\prime \prime }\left(\theta \right)= & 0.0348{\left[\frac{c_{p,g}k_{g}^{2}}{\mu_{g}}\rho_{g}g\left(\rho_{f}-\rho_{g}\right)\right]}^{0.549}{\left[0.414{\left(\Delta T_{\mathrm{MHF}}\right)}^{0.399}-0.112\right]}^{3.1217}\\ \; & \times \left[1+0.2\sin(1.1\theta )\right]\left[1+2.8e^{-0.36{\left(L_{c}/\lambda_{d}\right)}^{2}}\right]{\left(\frac{k_{\mathrm{Cu}}}{1+k_{\mathrm{Cu}}}\right)}^{0.07}. \end{array}$$

In this formula, *c*_*p*,*g*_, *k*_*g*_, *μ*_*g*_, and *ρ*_*g*_ denote, respectively, the specific heat at constant pressure, thermal conductivity, dynamic viscosity, and density of the saturated vapor. The multiplier containing *L*_*c*_/*λ*_*d*_ describes the effect of the minimum surface dimension [23] (Equations (18)–(21), pp. 11–12).

Transition boiling.

The transition-boiling (TB) branch connects the CHF and MHF points. Ahmad et al. [23] (Equations (22) and (23), pp. 11–12) define its superheat by

(7) $$\Delta T_{\mathrm{TB}}=\Delta T_{\mathrm{CHF}}\left(\theta \right)+\phi \cdot \left(\Delta T_{\mathrm{MHF}}-\Delta T_{\mathrm{CHF}}\left(\theta \right)\right),$$

where the dimensionless weighting parameter

(8) $$\phi ={\left|\frac{q_{\mathrm{TB}}^{\prime \prime }-q_{\mathrm{CHF}}^{\prime \prime }\left(\theta \right)}{q_{\mathrm{MHF}}^{\prime \prime }\left(\theta \right)-q_{\mathrm{CHF}}^{\prime \prime }\left(\theta \right)}\right|}^{0.8}$$

varies from 0 at the CHF point to 1 at the MHF point. After solving Equations (7) and (8) for $q_{\mathrm{TB}}^{\prime \prime }$, we obtain

$$q_{\mathrm{TB}}^{\prime \prime }\left(\Delta T;\theta \right)=q_{\mathrm{CHF}}^{\prime \prime }\left(\theta \right)+\left(q_{\mathrm{MHF}}^{\prime \prime }\left(\theta \right)-q_{\mathrm{CHF}}^{\prime \prime }\left(\theta \right)\right){\left(\frac{\Delta T-\Delta T_{\mathrm{CHF}}\left(\theta \right)}{\Delta T_{\mathrm{MHF}}-\Delta T_{\mathrm{CHF}}\left(\theta \right)}\right)}^{5/4},$$

for

$$\Delta T_{\mathrm{CHF}}\left(\theta \right)\leq \Delta T\leq \Delta T_{\mathrm{MHF}}.$$

Film boiling.

After substituting

$$T=T_{\mathrm{sat}}+\Delta T$$

into the updated correlation [23] (Equation (31), p. 17), the heat transfer coefficient in the film-boiling regime takes the form

(9) $$\begin{array}{cc} h_{\mathrm{FB}}\left(\Delta T;\theta \right)= & \left[0.148+0.052\sin\theta \right]\frac{k_{g}}{L_{b}}{\left[Ra_{g}\frac{h_{fg}+0.46c_{p,g}\Delta T}{c_{p,g}\Delta T}\right]}^{0.33}\left[1+2e^{-0.36{\left(L_{c}/\lambda_{d}\right)}^{2}}\right]\\ \; & +\left[3.5-0.55\sin\theta \right]\sigma_{\mathrm{SB}}\frac{{\left(T_{\mathrm{sat}}+\Delta T\right)}^{4}-T_{\mathrm{sat}}^{4}}{\Delta T}. \end{array}$$

The multipliers

0.148+0.052sinθand3.5−0.55sinθ

describe the effect of surface orientation on heat transport through the vapor film and on the radiative component of the correlation, respectively. The orientation dependence used in the FB correlation was validated for 0° ≤ *θ* ≤ 90°. For *θ* = 180°, we use its extrapolation, treating this extension as a modeling assumption. In Equation (9), *σ*_SB_ is the Stefan–Boltzmann constant:

$$\sigma_{\mathrm{SB}}=5.67\times 10^{-8}\;W/\left(m^{2}\;K^{4}\right).$$

The quantity *L*_*b*_, referred to as the Laplace length, is defined by

$$L_{b}={\left[\frac{\sigma }{g(\rho_{f}-\rho_{g})}\right]}^{1/2},$$

whereas the vapor Rayleigh number is defined by

$$Ra_{g}=\frac{L_{b}^{3}\rho_{g}\left(\rho_{f}-\rho_{g}\right)g}{\mu_{g}^{2}}\left(\frac{\mu_{g}c_{p,g}}{k_{g}}\right).$$

The relations defining *L*_*b*_ and *Ra*_*g*_ are given by Ahmad et al. [23] (Equations (27) and (28), p. 13). The first term of Equation (9) describes heat transport through the vapor film, whereas the second represents radiative heat transfer. The values of *L*_*b*_ and *Ra*_*g*_ are determined using the thermophysical properties adopted for the system considered. The multiplier

$$1+2e^{-0.36{\left(L_{c}/\lambda_{d}\right)}^{2}}$$

accounts for the effect of the minimum surface dimension.

The authors did not introduce a surface-roughness correction into the FB correlation, because its influence is limited by the presence of a continuous vapor layer. They also did not include a material correction because the effect of material is negligible at the high temperatures characteristic of the upper part of the FB regime, nor a subcooling correction, for which they did not have sufficient experimental data [23] (Sections 2.8.3–2.8.5, pp. 14–17).

Based on the heat transfer coefficient *h*_FB_(Δ*T*; *θ*), defined by Equation (9), the heat flux in the film-boiling regime is determined as

$$q_{\mathrm{FB}}^{\prime \prime }\left(\Delta T;\theta \right)=h_{\mathrm{FB}}\left(\Delta T;\theta \right)\;\Delta T.$$

Blending of the MHF point with the FB branch.

Direct combination of independently developed TB and FB correlations may lead to a discontinuity in the heat-flux value at the MHF point. Ahmad et al. remove this discontinuity by introducing a blending function between the MHF point and the point on the proper FB branch corresponding to $1.5q_{\mathrm{MHF}}^{\prime \prime }\left(\theta \right)$ [22] (Section 2.8, Equations (17) and (18), p. 6). Let Δ*T*_FB,1.5_(*θ*) denote the solution of

$$q_{\mathrm{FB}}^{\prime \prime }\left(\Delta T_{\mathrm{FB},1.5}\left(\theta \right);\theta \right)=1.5q_{\mathrm{MHF}}^{\prime \prime }\left(\theta \right),$$

located on the proper FB branch, i.e., satisfying

$$\Delta T_{\mathrm{FB},1.5}\left(\theta \right)>\Delta T_{\mathrm{MHF}}.$$

For the heat flux varying over the interval

$$q_{\mathrm{MHF}}^{\prime \prime }\left(\theta \right)\leq q^{\prime \prime }\leq 1.5q_{\mathrm{MHF}}^{\prime \prime }\left(\theta \right)$$

the source relation defines the superheat Δ*T*_blend_ as a function of $q^{\prime \prime }$:

(10) $$\Delta T_{\mathrm{blend}}\left(q^{\prime \prime };\theta \right)=\Delta T_{\mathrm{MHF}}+{\left|\frac{q^{\prime \prime }-q_{\mathrm{MHF}}^{\prime \prime }\left(\theta \right)}{0.5q_{\mathrm{MHF}}^{\prime \prime }\left(\theta \right)}\right|}^{1/2}\left(\Delta T_{\mathrm{FB},1.5}\left(\theta \right)-\Delta T_{\mathrm{MHF}}\right).$$

This continuous function takes values in the interval

$$\Delta T_{\mathrm{MHF}}\leq \Delta T_{\mathrm{blend}}\leq \Delta T_{\mathrm{FB},1.5}\left(\theta \right),$$

with $\Delta T_{\mathrm{blend}}\left(q_{\mathrm{MHF}}^{\prime \prime }\left(\theta \right);\theta \right)=\Delta T_{\mathrm{MHF}}$ and $\Delta T_{\mathrm{blend}}\left(1.5q_{\mathrm{MHF}}^{\prime \prime }\left(\theta \right);\theta \right)=\Delta T_{\mathrm{FB},1.5}\left(\theta \right)$. By solving Equation (10) for $q^{\prime \prime }$ we get its inverse function

(11) $$q_{\mathrm{blend}}^{\prime \prime }\left(\Delta T;\theta \right)=q_{\mathrm{MHF}}^{\prime \prime }\left(\theta \right)\left[1+\frac{1}{2}{\left(\frac{\Delta T-\Delta T_{\mathrm{MHF}}}{\Delta T_{\mathrm{FB},1.5}\left(\theta \right)-\Delta T_{\mathrm{MHF}}}\right)}^{2}\right],$$

for

$$\Delta T_{\mathrm{MHF}}\leq \Delta T\leq \Delta T_{\mathrm{FB},1.5}\left(\theta \right).$$

The exponent 1/2, the level $1.5q_{\mathrm{MHF}}^{\prime \prime }\left(\theta \right)$, and the difference $0.5q_{\mathrm{MHF}}^{\prime \prime }\left(\theta \right)$ are elements of the empirical blending function of Ahmad et al.

## 3. Boiling Curve for a CNT Buckypaper Layer on Cu

The CNT buckypaper–Cu boiling curve consists of the following segments:

(12) $$q_{\mathrm{CNT}}^{\prime \prime }\left(\Delta T;\theta \right)=\left\{\begin{array}{cc} q_{\mathrm{NC}–\mathrm{NB},\mathrm{CNT}}^{\prime \prime }\left(\Delta T;\theta \right),\; & 0\leq \Delta T\leq \Delta T_{\mathrm{CHF},\mathrm{CNT}}\left(\theta \right),\\ q_{\mathrm{TB},\mathrm{CNT}}^{\prime \prime }\left(\Delta T;\theta \right),\; & \Delta T_{\mathrm{CHF},\mathrm{CNT}}\left(\theta \right)<\Delta T\leq \Delta T_{\mathrm{MHF},\mathrm{CNT}},\\ q_{\mathrm{blend},\mathrm{CNT}}^{\prime \prime }\left(\Delta T;\theta \right),\; & \Delta T_{\mathrm{MHF},\mathrm{CNT}}<\Delta T\leq \Delta T_{J,\mathrm{CNT}}\left(\theta \right),\\ q_{\mathrm{Cu}}^{\prime \prime }\left(\Delta T;\theta \right),\; & \Delta T>\Delta T_{J,\mathrm{CNT}}\left(\theta \right). \end{array}\right.$$

The characteristic points delimiting these segments are denoted by

$${\mathrm{CHF}}_{\mathrm{CNT}}=\left(\Delta T_{\mathrm{CHF},\mathrm{CNT}}\left(\theta \right),q_{\mathrm{CHF},\mathrm{CNT}}^{\prime \prime }\left(\theta \right)\right),$$

$${\mathrm{MHF}}_{\mathrm{CNT}}=\left(\Delta T_{\mathrm{MHF},\mathrm{CNT}},q_{\mathrm{MHF},\mathrm{CNT}}^{\prime \prime }\left(\theta \right)\right),$$

and

$$J_{\mathrm{CNT}}=\left(\Delta T_{J,\mathrm{CNT}}\left(\theta \right),q_{J,\mathrm{CNT}}^{\prime \prime }\left(\theta \right)\right),$$

where J_CNT_ denotes the junction point between the transformed blending segment and the stable FB branch. In the following, we show how the individual branches in Equation (12) and the characteristic points CHF_CNT_, MHF_CNT_, and J_CNT_ are obtained from the corresponding branches and characteristic points of the reference Cu boiling curve.

### 3.1. Literature Evidence Concerning the CNT Layer on Cu and Porous Surfaces

The literature basis for transforming the reference Cu boiling curve into a curve corresponding to a CNT buckypaper layer deposited on a Cu surface and in contact with LN_2_ consists of two groups of studies. The first covers CNT coatings deposited on metallic surfaces and provides direct evidence concerning nucleate boiling and the CHF level. The second concerns capillary-porous surfaces investigated during LN_2_ boiling and indicates that layer morphology may also affect the shape of the curve, hysteresis, and the development of crisis phenomena. None of the cited studies, however, present a complete boiling curve for the CNT buckypaper–Cu system in LN_2_. Because of the scope of the available data, the operator parameters are determined primarily on the basis of studies conducted for horizontal upward-facing surfaces. As a first approximation, however, we assume that they describe the relative influence of the buckypaper layer and can also be applied to reference Cu curves determined for other surface orientations. This assumption is a modeling hypothesis and requires experimental verification in the future.

The transformation of the reference Cu boiling curve for a fixed orientation *θ* will be controlled by the following parameters: *η*_*i*_, *ξ*_*i*_, *i* = 1, 2. For clarity, we add the subscript Cu to the characteristic-point coordinates of the reference curve defined in the preceding section.

(13) $$\eta_{1}=\frac{\Delta T_{\mathrm{CHF},\mathrm{CNT}}\left(\theta \right)}{\Delta T_{\mathrm{CHF},\mathrm{Cu}}\left(\theta \right)},\;\eta_{2}=\frac{q_{\mathrm{CHF},\mathrm{CNT}}^{\prime \prime }\left(\theta \right)}{q_{\mathrm{CHF},\mathrm{Cu}}^{\prime \prime }\left(\theta \right)},\;\xi_{1}=\frac{\Delta T_{\mathrm{MHF},\mathrm{CNT}}}{\Delta T_{\mathrm{MHF},\mathrm{Cu}}},\;\xi_{2}=\frac{q_{\mathrm{MHF},\mathrm{CNT}}^{\prime \prime }\left(\theta \right)}{q_{\mathrm{MHF},\mathrm{Cu}}^{\prime \prime }\left(\theta \right)}.$$

In the adopted correlation, the superheat at the MHF point does not depend on surface orientation; therefore, the argument *θ* appears for the ordinate of this point but not for its abscissa. The parameters *η*_*i*_ and *ξ*_*i*_ are not written as functions of angle because, according to the adopted hypothesis, their values remain unchanged for the surface orientations considered.

Nanoporous MWCNT coating on Cu.

Mao, Hu, and Zhou [5] investigated a nanoporous MWCNT coating fabricated on a smooth Cu surface by self-assembly during saturated pool boiling of distilled water at atmospheric pressure. Relative to the uncoated Cu surface, they reported an increase of 86.4% in critical heat flux and 72.6% in the maximum heat transfer coefficient [5] (p. 184). The reported CHF increase gives

(14) $$\eta_{2}^{\left(\mathrm{Mao}\right)}=\frac{q_{\mathrm{CHF},\mathrm{MWCNT}}^{\prime \prime }}{q_{\mathrm{CHF},\mathrm{Cu}}^{\prime \prime }}=1.864.$$

The numerator and denominator refer to the coated and uncoated surfaces investigated by Mao et al. We use their second result to calibrate $\eta_{1}^{\left(\mathrm{Mao}\right)}$. For this purpose, we assume that the NC–NB segment is scaled by $\eta_{1}^{\left(\mathrm{Mao}\right)}$ along the superheat axis and by $\eta_{2}^{\left(\mathrm{Mao}\right)}$ along the heat-flux axis. Because $h=q^{\prime \prime }/\Delta T$, this transformation scales the heat transfer coefficient at corresponding points by $\eta_{2}^{\left(\mathrm{Mao}\right)}/\eta_{1}^{\left(\mathrm{Mao}\right)}$. Let *h*_max,MWCNT_ and *h*_max,Cu_ denote the maximum heat transfer coefficients on the NC–NB segments of the coated and uncoated surfaces, respectively. The reported 72.6% increase therefore gives the calibration condition

$$\frac{\eta_{2}^{\left(\mathrm{Mao}\right)}}{\eta_{1}^{\left(\mathrm{Mao}\right)}}=\frac{h_{\max,\mathrm{MWCNT}}}{h_{\max,\mathrm{Cu}}}=1.726.$$

Combining this condition with Equation (14), we obtain

$$\eta_{1}^{\left(\mathrm{Mao}\right)}=\frac{1.864}{1.726}≃1.080.$$

Thus, $\eta_{2}^{\left(\mathrm{Mao}\right)}$ follows directly from the measured CHF increase, whereas $\eta_{1}^{\left(\mathrm{Mao}\right)}$ is calibrated under the assumed scaling of the NC–NB segment; it is not a directly measured CHF superheat ratio.

The authors associated the improvement with increased wettability, roughness, capillary liquid wicking, and the number of active nucleation sites, which facilitated liquid replenishment and increased the bubble-departure frequency [5] (p. 184).

Multilayer PEI–MWCNT coatings on a metallic surface.

Lee et al. [6] investigated coatings fabricated using a layer-by-layer (LbL) method from PEI and MWCNT, deposited on stainless steel SS316. Coatings containing 10, 20, and 40 bilayers were fabricated, and the reference surfaces were uncoated SS316 steel and an MWCNT coating obtained by vacuum filtration [6] (p. 607). All LbL coatings increased the heat transfer coefficient and CHF relative to the uncoated substrate. The coating containing 20 bilayers provided the greatest improvement in the heat transfer coefficient, whereas the coating containing 40 bilayers achieved the largest CHF enhancement factor, approximately 147% [6] (p. 607).

Analogously to the parameter *η*_2_ defined in Equation (13), the result of Lee et al. can be written as the ratio

$$\eta_{2,\mathrm{SS}316}^{\left(\mathrm{Lee}\right)}\equiv \frac{q_{\mathrm{CHF},\mathrm{PEI}-\mathrm{MWCNT}}^{\prime \prime }}{q_{\mathrm{CHF},\mathrm{SS}316}^{\prime \prime }}≃1.47.$$

The additional subscript SS316 indicates that the reference was the steel surface. The ratio $\eta_{2,\mathrm{SS}316}^{\left(\mathrm{Lee}\right)}$ is therefore not a direct value of the parameter *η*_2_ for the CNT–Cu system considered, but provides additional evidence supporting *η*_2_ > 1.

The authors associated the improved heat transfer with the presence of nanocavities and an internal nanoporous structure. Compared with the coating obtained by vacuum filtration, a denser MWCNT network formed through electrostatic bonding and the hydrophilic nature of PEI were also important [6] (p. 607). These results show that the effect of the CNT layer depends on its architecture, thickness, porosity, manner of nanotube bonding, and wettability, and that the configuration providing the greatest improvement in the heat transfer coefficient need not simultaneously be the configuration providing the highest CHF.

Capillary-porous coatings during LN_2_ boiling.

Surtaev et al. [31] investigated LN_2_ boiling on coatings produced by plasma spraying, with thicknesses of 400 and 1390μm and porosity reaching 80%. The greatest heat-transfer enhancement, of the order of 300% relative to the smooth surface, was obtained at low heat fluxes for the thicker coating [31] (pp. 146, 147). For the 400μm coating, hysteresis was observed, and coating morphology also affected the development of the boiling crisis. Under stepwise loading, rapid transition to film boiling on the coated surfaces occurred at heat fluxes approximately twice as high as the CHF determined under steady-state conditions [31] (pp. 146, 147). These results provide qualitative evidence supporting changes not only in NB and CHF but also in the transition branch and the conditions for transition to FB.

Microstructured porous coatings on copper heaters in LN_2_.

Kuznetsov and Pavlenko [32] investigated capillary-porous coatings produced by directional plasma spraying on tubular copper heaters with a diameter of 16mm. The experiments were conducted during nitrogen boiling under steady heat generation. The coatings increased CHF relative to the smooth heater, while enhancing the heat transfer coefficient by up to a factor of 3.5 at low heat fluxes [32] (p. 481).

Analysis of high-speed image recordings indicated that, at low heat fluxes, the improvement in heat transfer was associated with substantial activation of stably operating nucleation sites. As the load increased, intense heat transport within the macrolayer in local spaces between the ridges of the structure became increasingly important [32] (p. 481). The authors also observed a reduction in the slope of the boiling curves for modified heaters in the pre-critical range. This phenomenon was associated with pulsating behavior of the phase boundaries and substantial surface-temperature fluctuations [32] (p. 481).

These results provide qualitative evidence that a porous surface may affect not only CHF and heat-transfer intensity but also the course of the curve in the pre-critical range. The study did not, however, concern CNT or buckypaper and does not provide direct values of the parameters *η*_1_, *η*_2_, *ξ*_1_, or *ξ*_2_ for the CNT–Cu system considered in LN_2_.

Additional evidence concerning the influence of porous structure in the film-boiling regime is provided by the results of Hu et al. [33] (Figures 5, 7 and 10). In experiments with liquid nitrogen, they showed that, in the stable FB regime, the heat fluxes for the nanoporous surface and the reference surface are similar, and the observed vapor-film structures have a similar character. The authors relate this to the fact that, in stable film boiling, the heating surface is separated from the liquid by a vapor layer, as a result of which the influence of surface properties and microstructure on the heat-transfer mechanism is substantially reduced. These results do not directly concern CNT buckypaper on Cu; therefore, they are used only as qualitative evidence supporting the assumption that the influence of the BP layer rapidly vanishes after transition to the stable FB regime.

### 3.2. Parameter Ranges and Transformation Scenarios

On the basis of the literature evidence discussed in the preceding subsection, we determine the direction and approximate scale of possible changes in the coordinates of the CHF and MHF points relative to the reference Cu boiling curve. For the MHF point, available results for micro- and nanostructured surfaces in LN_2_ provide evidence supporting the possibility of shifting its coordinates, but they do not provide direct parameter values for the CNT buckypaper–Cu system considered. According to the adopted hypothesis, the parameter ranges and their values in the individual scenarios remain independent of surface orientation. These ranges are not directly measured properties of the CNT buckypaper–Cu system in LN_2_ but define the domain of values adopted for the analysis. Within this domain, two parameter sets are defined: a moderately optimistic scenario $S_{1}$ and an optimistic scenario $S_{2}$. A value of 1 indicates no change in the corresponding coordinate of the characteristic point relative to the reference Cu curve. For the parameters describing the change in the CHF point, we adopt

$$\eta_{1}\in \left[1.0,\;1.1\right],\;\eta_{2}\in \left[1.0,\;1.864\right],$$

where *η*_1_ describes the change in superheat at the CHF point, whereas *η*_2_ describes the change in critical heat flux. For the parameters describing the change in the MHF point, we adopt

$$\xi_{1}\in \left[1.0,\;1.2\right],\;\xi_{2}\in \left[1.0,\;1.2\right],$$

where *ξ*_1_ describes the change in superheat at the MHF point, whereas *ξ*_2_ describes the change in minimum heat flux. The adopted ranges of *ξ*_1_ and *ξ*_2_ have the character of a phenomenological modeling assumption. For the parameter *ξ*_1_, quantitative evidence concerning the direction and possible magnitude of the change is provided by the results of Hu et al. [33] (Figure 10 and the discussion therein). In quenching experiments in LN_2_, the Leidenfrost point, corresponding to the minimum heat flux, occurred at a superheat of approximately 42K for the reference surface and approximately 82K for the nanoporous surface. This corresponds to a change in the abscissa of the MHF point by a factor of approximately 1.95, i.e., substantially larger than the value *ξ*_1_ ≤ 1.2 adopted in the present work. Because the study of Hu et al. concerned a nanoporous anodic aluminum oxide (AAO) surface on aluminum and a quenching process, this value is not transferred directly to the CNT buckypaper–Cu system; rather, it is treated as evidence indicating that the adopted *ξ*_1_ range constitutes a conservative first-approximation scenario. For the parameter *ξ*_2_, quantitative evidence is provided by the results of Sathyamurthi et al. [34] (Table 1, p. 071501-8). In saturated pool boiling of PF-5060, the heat flux at the MHF point for a Si surface coated with a Type-B MWCNT layer was $5.5\;W/{\mathrm{cm}}^{2}$, compared with $3.4\;W/{\mathrm{cm}}^{2}$ for the uncoated Si surface, corresponding to an increase of approximately 62%. Because these experiments concerned MWCNT on a Si substrate and a different working fluid, the reported values are not transferred directly to the CNT buckypaper–Cu system in LN_2_. The adopted range $\xi_{2}\in \left[1.0,\;1.2\right]$ is therefore treated as a conservative, phenomenological scenario assumption consistent with the experimentally observed direction of change in the MHF point. In summary, we consider the following two scenarios.

Scenario 1: moderately optimistic.

$$S_{1}:\;\eta_{1}=1.05,\;\eta_{2}=1.50,\;\xi_{1}=1.10,\;\xi_{2}=1.10.$$

Scenario 2: optimistic.

$$S_{2}:\;\eta_{1}=1.10,\;\eta_{2}=1.80,\;\xi_{1}=1.20,\;\xi_{2}=1.20.$$

## 4. Operator-Based Construction of the Boiling Curve for a CNT Buckypaper Layer on Cu

### 4.1. Transformation Operator for the Reference Curve

**Figure 2:**
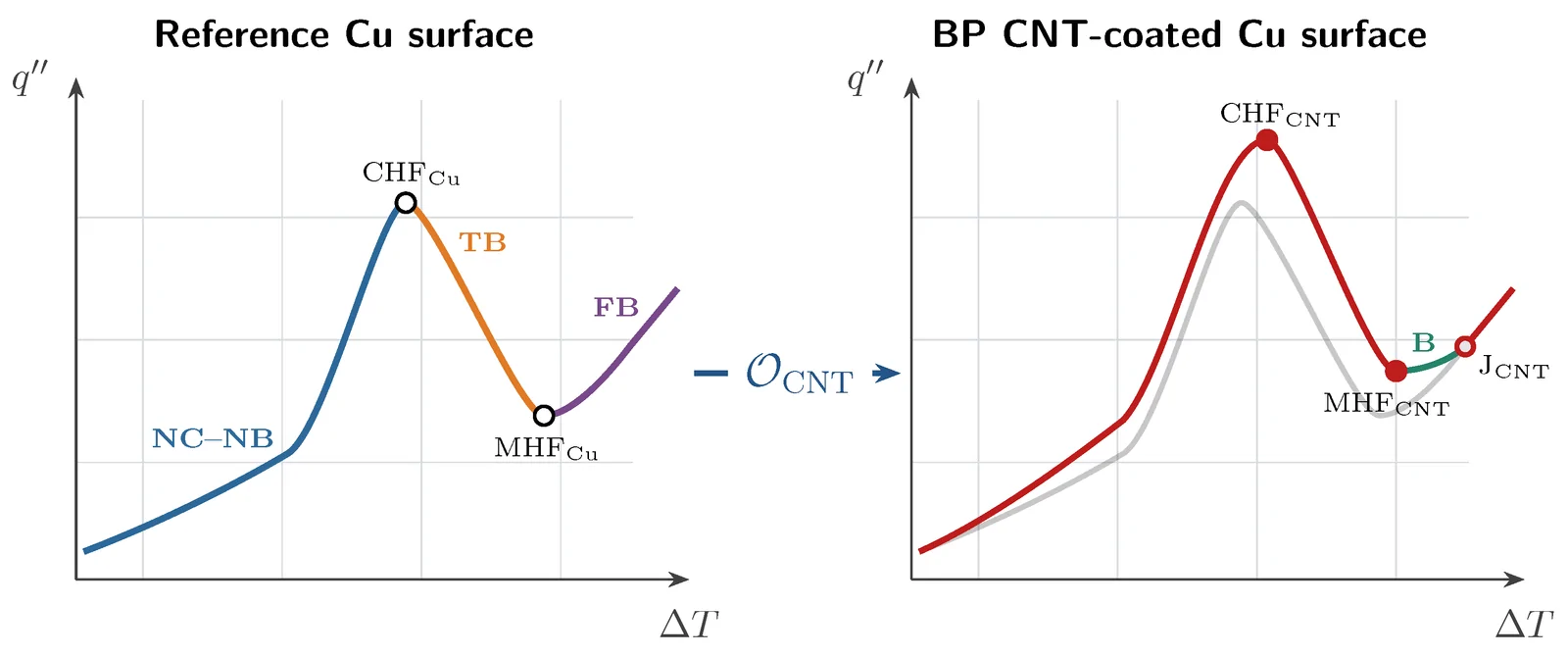
Schematic representation of the operator-based transformation of the reference Cu–LN_2_ boiling curve into a scenario boiling curve for a Cu surface coated with CNT buckypaper. NC–NB—natural convection and nucleate boiling; TB—transition-boiling; FB—film boiling; B—blending segment; J_CNT_—junction point.

For a fixed surface orientation *θ*, the curve $q_{\mathrm{CNT}}^{\prime \prime }\left(\Delta T;\theta \right)$ is formally defined as

$$q_{\mathrm{CNT}}^{\prime \prime }\left(\Delta T;\theta \right)=O_{\mathrm{CNT}}\;\left[q_{\mathrm{Cu}}^{\prime \prime }\left(\;\cdot \;;\theta \right)\right]\left(\Delta T;\eta ,\xi \right),$$

where

$$\eta =\left(\eta_{1},\eta_{2}\right),\;\xi =\left(\xi_{1},\xi_{2}\right)$$

are parameter vectors defined by Equation (13). The vector ***η*** describes the change in the coordinates of the CHF point, whereas the vector ***ξ*** describes the change in the coordinates of the MHF point. According to the adopted hypothesis, the component values of these vectors remain independent of surface orientation. The operator acts on the curve $q_{\mathrm{Cu}}^{\prime \prime }\left(\Delta T;\theta \right)$, determined for the same surface orientation, modifying its characteristic points while preserving the normalized shape of the individual branches of the Cu boiling curve. It acts separately on each of the identified curve segments, while preserving continuity of the heat-flux values at their junctions. The general action of the operator is illustrated schematically in Figure 2.

### 4.2. Branch Transformation Operator

Let

f:[a,b]→R,f(a)≠f(b),

be a continuous and monotonic function. We introduce two vectors

$$\zeta =\left(\zeta_{1},\zeta_{2}\right),\;\kappa =\left(\kappa_{1},\kappa_{2}\right),$$

which define the transformations of the initial and final points of the graph of *f*, respectively:

$$\left(a,f(a)\right)⟼\left(\zeta_{1}a,\zeta_{2}f\left(a\right)\right),\;\left(b,f(b)\right)⟼\left(\kappa_{1}b,\kappa_{2}f\left(b\right)\right).$$

We seek to transform the function *f* into a function *g* with the following properties:

- The domain of *g* is the interval [*ζ*_1_*a*, *κ*_1_*b*];
- The values of *g* at the endpoints satisfy
  $$g\left(\zeta_{1}a\right)=\zeta_{2}f\left(a\right),\;g\left(\kappa_{1}b\right)=\kappa_{2}f\left(b\right);$$
- The function *g* preserves the direction of monotonicity and the normalized shape of *f*.

Such a function is obtained using the operator

(15) $$g\left(x\right)=T_{\zeta ,\kappa }\left[f\right]\left(x\right)=\zeta_{2}f\left(a\right)+\frac{\kappa_{2}f\left(b\right)-\zeta_{2}f\left(a\right)}{f(b)-f(a)}\left[f\;\left(a+\frac{b-a}{\kappa_{1}b-\zeta_{1}a}\left(x-\zeta_{1}a\right)\right)-f\left(a\right)\right],$$

for

$$\zeta_{1}a\leq x\leq \kappa_{1}b.$$

Under the assumptions

$$\kappa_{1}b>\zeta_{1}a,\;\frac{\kappa_{2}f\left(b\right)-\zeta_{2}f\left(a\right)}{f(b)-f(a)}>0$$

the function *g* preserves the direction of monotonicity and the normalized shape of *f*.

### 4.3. Transformation of the Branches of the Reference Curve

For clarity, we append Cu in the subscript of the branch labels introduced in Equation (2) when referring to segments of the reference curve.

NC–NB branches.

The segment of the reference Cu boiling curve comprising the natural-convection and nucleate-boiling branches is denoted by $q_{\mathrm{NC}–\mathrm{NB},\mathrm{Cu}}^{\prime \prime }$:

$$q_{\mathrm{NC}–\mathrm{NB},\mathrm{Cu}}^{\prime \prime }\left(\Delta T;\theta \right)=q_{\mathrm{Cu}}^{\prime \prime }\left(\Delta T;\theta \right),\;\Delta T_{L,4,\mathrm{Cu}}\leq \Delta T\leq \Delta T_{\mathrm{CHF},\mathrm{Cu}}\left(\theta \right).$$

To preserve the normalized shape of the entire NC–NB segment, the same coordinate transformation is applied to both of its endpoints:

$$\begin{array}{cc} \left(\Delta T_{L,4,\mathrm{Cu}},q_{\mathrm{Cu}}^{\prime \prime }\left(\Delta T_{L,4,\mathrm{Cu}};\theta \right)\right)\; & ⟼\left(\eta_{1}\Delta T_{L,4,\mathrm{Cu}},\eta_{2}q_{\mathrm{Cu}}^{\prime \prime }\left(\Delta T_{L,4,\mathrm{Cu}};\theta \right)\right),\\ \left(\Delta T_{\mathrm{CHF},\mathrm{Cu}}\left(\theta \right),q_{\mathrm{CHF},\mathrm{Cu}}^{\prime \prime }\left(\theta \right)\right)\; & ⟼\left(\eta_{1}\Delta T_{\mathrm{CHF},\mathrm{Cu}}\left(\theta \right),\eta_{2}q_{\mathrm{CHF},\mathrm{Cu}}^{\prime \prime }\left(\theta \right)\right). \end{array}$$

In operator (15), this corresponds to the choice ***ζ*** = ***κ*** = ***η*** = (*η*_1_, *η*_2_). 

After applying the operator, we obtain

(16) $$q_{\mathrm{NC}–\mathrm{NB},\mathrm{CNT}}^{\prime \prime }\left(\Delta T;\theta \right)=T_{\eta ,\eta }\left[q_{\mathrm{NC}–\mathrm{NB},\mathrm{Cu}}^{\prime \prime }\left(\;\cdot \;;\theta \right)\right]\left(\Delta T\right)=\eta_{2}\;q_{\mathrm{Cu}}^{\prime \prime }\left(\frac{\Delta T}{\eta_{1}};\theta \right),$$

for

$$\eta_{1}\Delta T_{L,4,\mathrm{Cu}}\leq \Delta T\leq \eta_{1}\Delta T_{\mathrm{CHF},\mathrm{Cu}}\left(\theta \right).$$

The ONB point is an internal point of the transformed segment; therefore, its position follows from the action of the operator:

$$\left(\Delta T_{\mathrm{ONB},\mathrm{Cu}}\left(\theta \right),q_{\mathrm{ONB},\mathrm{Cu}}^{\prime \prime }\left(\theta \right)\right)⟼\left(\eta_{1}\Delta T_{\mathrm{ONB},\mathrm{Cu}}\left(\theta \right),\eta_{2}q_{\mathrm{ONB},\mathrm{Cu}}^{\prime \prime }\left(\theta \right)\right).$$

The formal range of Equation (16) begins at the superheat *η*_1_Δ*T*_*L*,4,Cu_. The range

$$0\leq \Delta T<\eta_{1}\Delta T_{L,4,\mathrm{Cu}}$$

will be closed at the numerical implementation stage by a continuous linear extension of the curve between the point (0, 0) and the first point of the formal range of the NC–NB branch.

TB branch.

Recall that the reference TB branch coincides with the Cu boiling curve over the interval Δ*T*_CHF,Cu_(*θ*) ≤ Δ*T* ≤ Δ*T*_MHF,Cu_. The endpoints of the graph of this branch are transformed according to

$$\begin{array}{cc} \left(\Delta T_{\mathrm{CHF},\mathrm{Cu}}\left(\theta \right),q_{\mathrm{CHF},\mathrm{Cu}}^{\prime \prime }\left(\theta \right)\right)\; & ⟼\left(\eta_{1}\Delta T_{\mathrm{CHF},\mathrm{Cu}}\left(\theta \right),\eta_{2}q_{\mathrm{CHF},\mathrm{Cu}}^{\prime \prime }\left(\theta \right)\right),\\ \left(\Delta T_{\mathrm{MHF},\mathrm{Cu}},q_{\mathrm{MHF},\mathrm{Cu}}^{\prime \prime }\left(\theta \right)\right)\; & ⟼\left(\xi_{1}\Delta T_{\mathrm{MHF},\mathrm{Cu}},\xi_{2}q_{\mathrm{MHF},\mathrm{Cu}}^{\prime \prime }\left(\theta \right)\right). \end{array}$$

These endpoint mappings correspond to the following parameter choice in the branch transformation operator:

$$\zeta =\eta =\left(\eta_{1},\eta_{2}\right),\;\kappa =\xi =\left(\xi_{1},\xi_{2}\right).$$

After applying the operator, we obtain

$$\begin{array}{cc} q_{\mathrm{TB},\mathrm{CNT}}^{\prime \prime }\left(\Delta T;\theta \right)= & T_{\left(\eta_{1},\eta_{2}\right),\left(\xi_{1},\xi_{2}\right)}\left[q_{\mathrm{TB},\mathrm{Cu}}^{\prime \prime }\left(\;\cdot \;;\theta \right)\right]\left(\Delta T\right)\\ = & \eta_{2}q_{\mathrm{CHF},\mathrm{Cu}}^{\prime \prime }\left(\theta \right)+\frac{\xi_{2}q_{\mathrm{MHF},\mathrm{Cu}}^{\prime \prime }\left(\theta \right)-\eta_{2}q_{\mathrm{CHF},\mathrm{Cu}}^{\prime \prime }\left(\theta \right)}{q_{\mathrm{MHF},\mathrm{Cu}}^{\prime \prime }\left(\theta \right)-q_{\mathrm{CHF},\mathrm{Cu}}^{\prime \prime }\left(\theta \right)}\\ \times & \left[q_{\mathrm{TB},\mathrm{Cu}}^{\prime \prime }\left(\Delta T_{\mathrm{CHF},\mathrm{Cu}}\left(\theta \right)+\alpha \left(\theta \right)\cdot \left(\Delta T-\eta_{1}\Delta T_{\mathrm{CHF},\mathrm{Cu}}\left(\theta \right)\right);\theta \right)-q_{\mathrm{CHF},\mathrm{Cu}}^{\prime \prime }\left(\theta \right)\right], \end{array}$$

where

$$\alpha \left(\theta \right)=\frac{\Delta T_{\mathrm{MHF},\mathrm{Cu}}-\Delta T_{\mathrm{CHF},\mathrm{Cu}}\left(\theta \right)}{\xi_{1}\Delta T_{\mathrm{MHF},\mathrm{Cu}}-\eta_{1}\Delta T_{\mathrm{CHF},\mathrm{Cu}}\left(\theta \right)},$$

for

$$\eta_{1}\Delta T_{\mathrm{CHF},\mathrm{Cu}}\left(\theta \right)\leq \Delta T\leq \xi_{1}\Delta T_{\mathrm{MHF},\mathrm{Cu}}.$$

The transformed branch satisfies

$$\begin{array}{cc} q_{\mathrm{TB},\mathrm{CNT}}^{\prime \prime }\left(\eta_{1}\Delta T_{\mathrm{CHF},\mathrm{Cu}}\left(\theta \right);\theta \right)\; & =\eta_{2}q_{\mathrm{CHF},\mathrm{Cu}}^{\prime \prime }\left(\theta \right),\\ q_{\mathrm{TB},\mathrm{CNT}}^{\prime \prime }\left(\xi_{1}\Delta T_{\mathrm{MHF},\mathrm{Cu}};\theta \right)\; & =\xi_{2}q_{\mathrm{MHF},\mathrm{Cu}}^{\prime \prime }\left(\theta \right). \end{array}$$

FB branch.

The reference blending segment between the MHF point and the proper FB branch begins at the MHF point and ends at the junction point with the proper FB branch, for which

$$q_{\mathrm{Cu}}^{\prime \prime }\left(\Delta T_{\mathrm{FB},1.5}\left(\theta \right);\theta \right)=1.5q_{\mathrm{MHF},\mathrm{Cu}}^{\prime \prime }\left(\theta \right).$$

The new junction point between the transformed blending segment and the proper FB branch is determined on the reference Cu curve. Its abscissa has the form *ν*(*θ*)Δ*T*_FB,1.5_(*θ*), where *ν*(*θ*) is defined as the solution of the following equation corresponding to a point lying on the proper FB branch of the reference Cu curve:

$$q_{\mathrm{Cu}}^{\prime \prime }\left(\nu \left(\theta \right)\Delta T_{\mathrm{FB},1.5}\left(\theta \right);\theta \right)=1.5\xi_{2}q_{\mathrm{MHF},\mathrm{Cu}}^{\prime \prime }\left(\theta \right).$$

Thus, the coordinates of the junction point J_CNT_ in (12) are

$$\Delta T_{J,\mathrm{CNT}}\left(\theta \right)=\nu \left(\theta \right)\Delta T_{\mathrm{FB},1.5}\left(\theta \right),\;q_{J,\mathrm{CNT}}^{\prime \prime }\left(\theta \right)=1.5\xi_{2}q_{\mathrm{MHF},\mathrm{Cu}}^{\prime \prime }\left(\theta \right).$$

The endpoints of the blending segment are therefore transformed.

In operator (15), this corresponds to the choice

$$\zeta =\left(\xi_{1},\xi_{2}\right),\;\kappa =\left(\nu \left(\theta \right),\xi_{2}\right).$$

The transformed blending segment is defined using operator (15) as

$$q_{\mathrm{blend},\mathrm{CNT}}^{\prime \prime }\left(\Delta T;\theta \right)=T_{\left(\xi_{1},\xi_{2}\right),\left(\nu \left(\theta \right),\xi_{2}\right)}\left[q_{\mathrm{blend},\mathrm{Cu}}^{\prime \prime }\left(\;\cdot \;;\theta \right)\right]\left(\Delta T\right),$$

for

$$\xi_{1}\Delta T_{\mathrm{MHF},\mathrm{Cu}}\leq \Delta T\leq \nu \left(\theta \right)\Delta T_{\mathrm{FB},1.5}\left(\theta \right).$$

After substituting the explicit form of the function $q_{\mathrm{blend},\mathrm{Cu}}^{\prime \prime }\left(\Delta T;\theta \right)$ from Equation (11), we obtain

$$q_{\mathrm{blend},\mathrm{CNT}}^{\prime \prime }\left(\Delta T;\theta \right)=\xi_{2}q_{\mathrm{MHF},\mathrm{Cu}}^{\prime \prime }\left(\theta \right)\left[1+\frac{1}{2}{\left(\frac{\Delta T-\xi_{1}\Delta T_{\mathrm{MHF},\mathrm{Cu}}}{\nu \left(\theta \right)\Delta T_{\mathrm{FB},1.5}\left(\theta \right)-\xi_{1}\Delta T_{\mathrm{MHF},\mathrm{Cu}}}\right)}^{2}\right],$$

for

$$\xi_{1}\Delta T_{\mathrm{MHF},\mathrm{Cu}}\leq \Delta T\leq \nu \left(\theta \right)\Delta T_{\mathrm{FB},1.5}\left(\theta \right).$$

The transformed blending segment satisfies

$$\begin{array}{cc} q_{\mathrm{blend},\mathrm{CNT}}^{\prime \prime }\left(\xi_{1}\Delta T_{\mathrm{MHF},\mathrm{Cu}};\theta \right)\; & =\xi_{2}q_{\mathrm{MHF},\mathrm{Cu}}^{\prime \prime }\left(\theta \right),\\ q_{\mathrm{blend},\mathrm{CNT}}^{\prime \prime }\left(\nu \left(\theta \right)\Delta T_{\mathrm{FB},1.5}\left(\theta \right);\theta \right)\; & =1.5\xi_{2}q_{\mathrm{MHF},\mathrm{Cu}}^{\prime \prime }\left(\theta \right). \end{array}$$

From the condition defining *ν*,

$$q_{\mathrm{Cu}}^{\prime \prime }\left(\nu \left(\theta \right)\Delta T_{\mathrm{FB},1.5}\left(\theta \right);\theta \right)=1.5\xi_{2}q_{\mathrm{MHF},\mathrm{Cu}}^{\prime \prime }\left(\theta \right),$$

it follows that the end of the transformed blending segment lies on the proper FB branch of the reference Cu curve.

For larger values of superheat, in accordance with the adopted assumption that the influence of the CNT layer vanishes in the stable film-boiling regime, the reference FB branch is left unchanged.

The segment of the curve for the CNT layer comprising the MHF–FB blending segment and the proper FB branch is therefore defined as

$$q_{\mathrm{blend}–\mathrm{FB},\mathrm{CNT}}^{\prime \prime }\left(\Delta T;\theta \right)=\left\{\begin{array}{cc} q_{\mathrm{blend},\mathrm{CNT}}^{\prime \prime }\left(\Delta T;\theta \right),\; & \xi_{1}\Delta T_{\mathrm{MHF},\mathrm{Cu}}\leq \Delta T\leq \nu \left(\theta \right)\Delta T_{\mathrm{FB},1.5}\left(\theta \right),\\ q_{\mathrm{Cu}}^{\prime \prime }\left(\Delta T;\theta \right),\; & \Delta T>\nu \left(\theta \right)\Delta T_{\mathrm{FB},1.5}\left(\theta \right). \end{array}\right.$$

From the definition of *ν*, the values of the two parts at the new junction point are equal; therefore, the resulting curve remains continuous.

**Figure 3:**
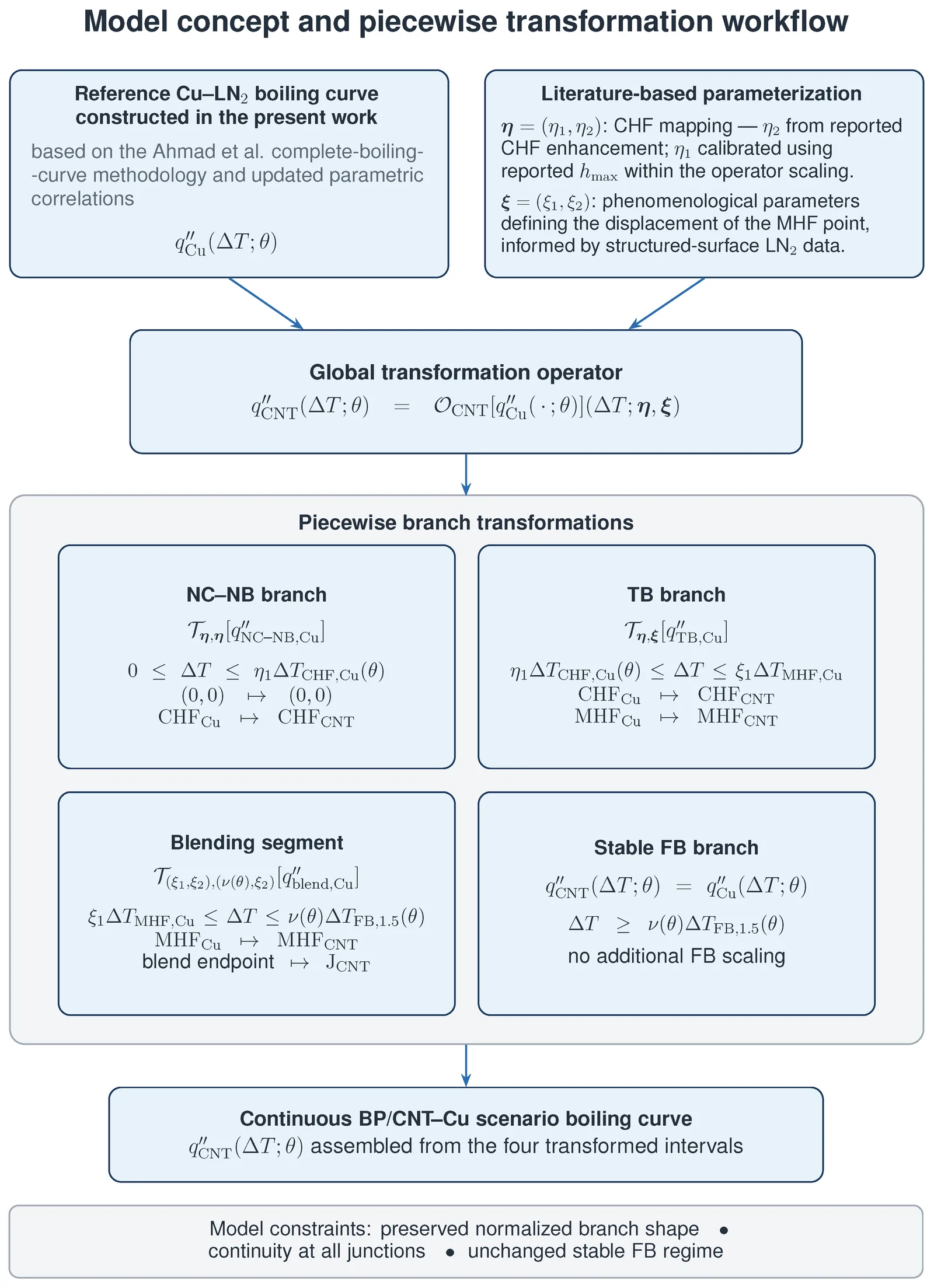
Workflow of the operator-based construction of the scenario boiling curve for a Cu surface coated with CNT buckypaper.

The complete operator-based construction procedure is summarized in Figure 3.

$$\begin{array}{cc} \left(\Delta T_{\mathrm{MHF},\mathrm{Cu}},q_{\mathrm{MHF},\mathrm{Cu}}^{\prime \prime }\left(\theta \right)\right)\; & ⟼\left(\xi_{1}\Delta T_{\mathrm{MHF},\mathrm{Cu}},\xi_{2}q_{\mathrm{MHF},\mathrm{Cu}}^{\prime \prime }\left(\theta \right)\right),\\ \left(\Delta T_{\mathrm{FB},1.5}\left(\theta \right),1.5q_{\mathrm{MHF},\mathrm{Cu}}^{\prime \prime }\left(\theta \right)\right)\; & ⟼\;J_{\mathrm{CNT}}. \end{array}$$

## 5. Numerical Implementation

The numerical implementation was carried out in MATLAB R2024b (Update 7) for the three surface orientations *θ* = 0°, 90°, and 180°, and for the two transformation scenarios $S_{1}$ and $S_{2}$ defined in Section 3.2. The system conditions, geometric and surface parameters, thermophysical properties of nitrogen, and scenario-specific transformation parameters used in the numerical calculations are summarized in Table 2.

The temperature-dependent liquid density required by the natural-convection correlation was evaluated using CoolProp through the MATLAB–Python interface (Python 3.11).

The reference Cu curve was assembled piecewise from the NC, NB, TB, MHF–FB blending, and proper FB branches. Because the formal range of the NC correlation begins at Δ*T*_*L*,4_ > 0, the interval between (0, 0) and the first point of this range was completed by a linear segment.

The implementation verifies the ordering of the characteristic points and tests the continuity of the transformed curve at the MHF point and at its junction with the unmodified Cu film-boiling branch. A relative tolerance of 10^−8^ was used in these continuity tests.

For plotting, the curves were evaluated at 1500 logarithmically spaced values of wall superheat over the range 0.05≤ΔT≤250K.

**Figure 4:**
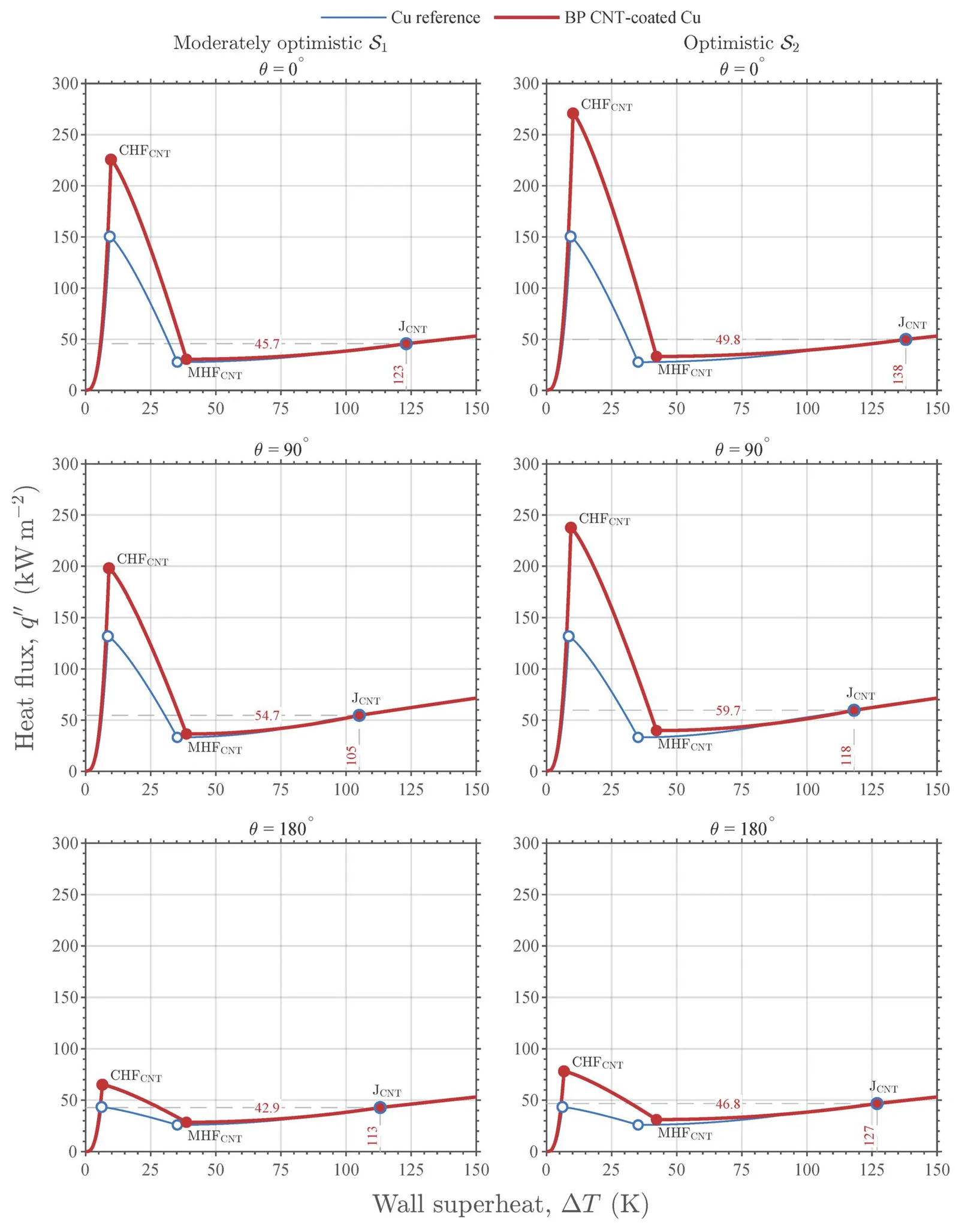
Comparison of the reference Cu–LN_2_ boiling curves and the transformed boiling curves for the CNT buckypaper layer (linear axes).

**Figure 5:**
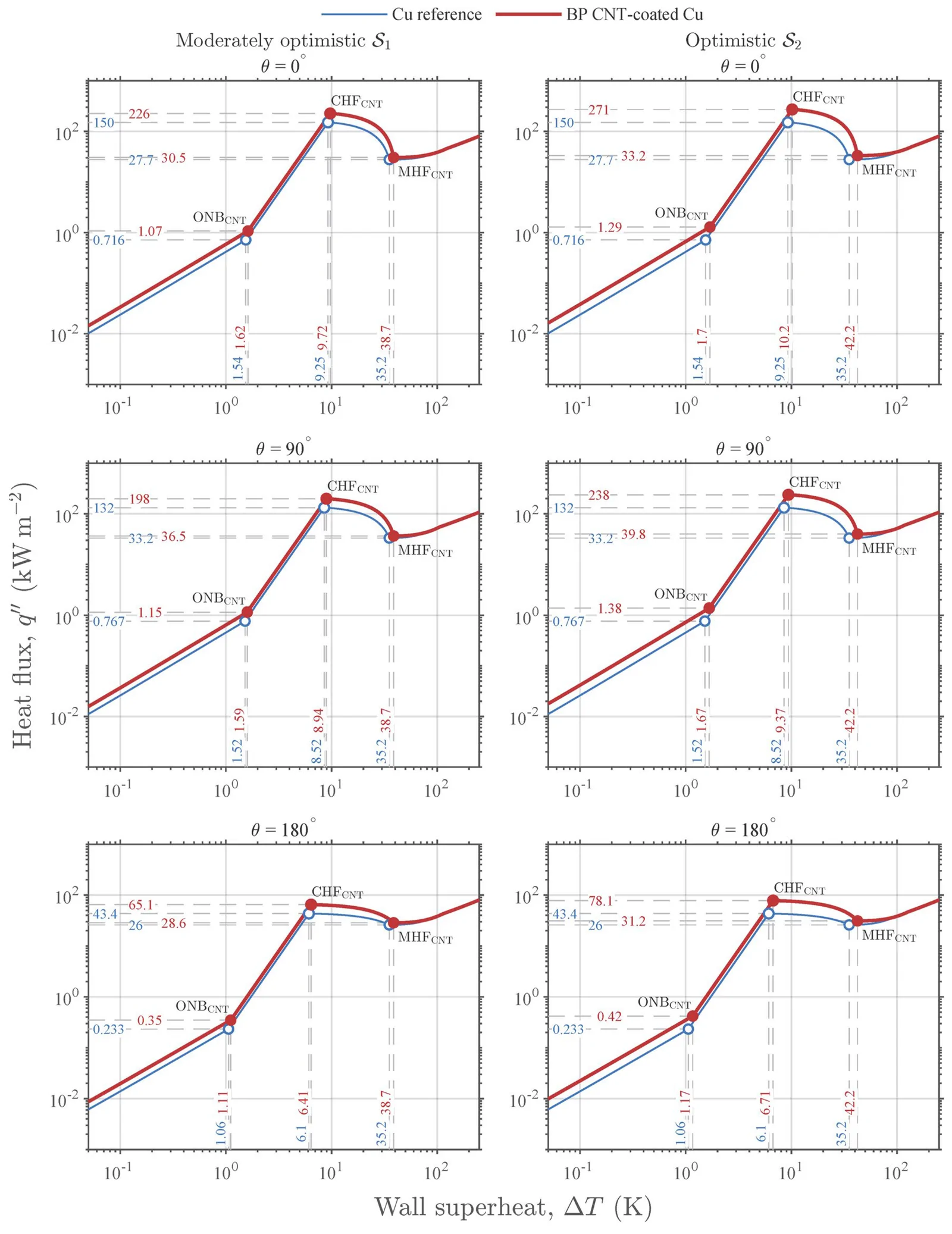
Comparison of the reference Cu–LN_2_ boiling curves and the transformed boiling curves for the CNT buckypaper layer (logarithmic axes).

Figure 4 and Figure 5 compare the reference Cu–LN_2_ boiling curves with the transformed curves obtained for Scenarios $S_{1}$ and $S_{2}$ at the three surface orientations considered.

The logarithmic representation shows the complete boiling characteristics over the full ranges of wall superheat and heat flux, whereas the linear representation provides a clearer view of the displacement of the characteristic points and the transition between the transformed blending segments and the unmodified film-boiling branches.

## 6. Results

A key aspect of this work is the reformulation of the methodology for constructing the complete Cu–LN_2_ boiling curve into a form suitable for direct numerical implementation. Wherever permitted by the source correlations, the relations describing the individual boiling regimes were expressed explicitly as functions of superheat. Numerical solution was retained for determining the Rayleigh-number thresholds and the points defined by intersections or junctions of the corresponding curve branches. The resulting representation preserves the parametric nature of the source correlations and can therefore be applied to different surface parameters and boiling conditions within their applicability ranges, rather than being restricted to a single system configuration.

CNT buckypaper was identified as a promising additional layer for enhancing heat removal from the REBCO tape. This choice was motivated by a combination of favorable properties: a porous structure that may enhance heat transfer to liquid nitrogen, high in-plane thermal conductivity that may promote lateral heat spreading, the possibility of tailoring the BP layer and the BP–Cu contact to limit the additional thermal resistance, and the technological feasibility of integrating buckypaper with a Cu surface. It is the combination of these properties, rather than any single one of them, that makes CNT buckypaper particularly suitable for the considered application. These considerations identify CNT buckypaper as a promising direction for further research on the cooling of superconducting tapes.

Because no complete experimental boiling characteristic is available for the BP–Cu–LN_2_ system, relevant findings from the literature were synthesized to establish a coherent basis for the proposed model. The analysis identified physically plausible trends in nucleate-boiling enhancement, shifts in the CHF and MHF points, and the transition to film boiling. On this basis, the direction and realistic ranges of changes in the key elements of the complete boiling curve were determined, providing the basis for constructing scenario-based curves for a CNT buckypaper layer on Cu.

On this basis, an operator-based procedure was developed to construct a complete boiling characteristic that is both physically and mathematically consistent, while keeping the underlying assumptions explicit. The procedure allows for subsequent calibration: once experimental data for the BP–Cu–LN_2_ system become available, the scenario-based parameter values can be replaced with experimentally determined values without modifying the structure of the operator. The proposed methodology can also be adapted to other modified surfaces and related problems for which complete experimental data are unavailable, but sufficient evidence exists to define a physically plausible transformation of a reference characteristic.

The operator modifies the individual segments of the curve according to the role of the characteristic points bounding each boiling regime, while the proper FB branch remains unchanged at higher superheats. This preserves the character of the boiling process and constitutes a key advantage over directly transferring individual experimental results to a different material–fluid system or scaling the entire curve by a single factor.

## 7. Discussion

The main limitation of the proposed model is the lack of complete experimental data for the BP–Cu–LN_2_ system. The available data on the effects of CNT layers were obtained for different substrates, working fluids, geometries, and experimental conditions. Consequently, the adopted ranges of the transformation parameters are scenario-based. In particular, experimental validation is required for the parameter values, their assumed independence from surface orientation, and the assumption that the influence of the CNT layer vanishes in the stable film-boiling regime.

In practice, quantitative verification of the cooling-improvement condition given by Equation (1) also requires reliable values of the Cu–BP contact resistance and the through-thickness thermal conductivity of the BP layer. The full anisotropic heat transport within the BP layer is not included in the present boiling-curve operator and will be addressed by developing an anisotropic thermal-conduction model in a subsequent study.

## 8. Conclusions

The proposed construction provides a way to incorporate incomplete evidence on surface modification into a complete boiling characteristic. Its main contribution is an operator that relates changes in individual boiling regimes to shifts of their characteristic points while maintaining continuity between the branches. This allows different assumptions concerning the BP layer to be represented within a common mathematical structure, rather than by applying a single enhancement factor to the entire reference curve.

The resulting characteristics provide boundary conditions for investigating how assumed changes in boiling behavior affect thermal and electrothermal responses of LN_2_-cooled systems. At the present stage, they support scenario comparisons rather than quantitative predictions of the cooling performance of a particular BP–Cu assembly. In particular, enhanced boiling at the BP–LN_2_ interface does not by itself establish improved cooling of the underlying Cu surface, because the thermal resistance of the BP layer and its contact with Cu must also be taken into account.

Further development requires experimental calibration for the BP–Cu–LN_2_ system and coupling of the boiling characteristic with an anisotropic thermal-conduction model of the BP layer. Measurements should also test the assumed orientation independence of the transformation parameters and the treatment of stable film boiling. Experimental parameter values can then replace the scenario-based values without changing the structure of the operator.

More broadly, the same approach may be adapted to other modified surfaces for which a reference boiling curve is available but experimental information on the modified system remains incomplete. For superconducting applications, it provides a basis for assessing whether potential improvements in interfacial boiling translate into lower conductor temperatures and improved thermal recovery.

## Figures and Tables

**Table 1:** Natural-convection correlation coefficients for the orientations considered by Ahmad et al. [23] (Table 1, p. 4).

θ	*C*	*n*	Applicability Range
0°	0.54	1/4	10^4^ ≤ *Ra*_*L*_ ≤ 10^7^, *Pr*_*l*_ ≥ 0.7
0°	0.15	1/3	10^7^ ≤ *Ra*_*L*_ ≤ 10^11^
90°	0.59	1/4	10^4^ ≤ *Ra*_*L*_ ≤ 10^9^
90°	0.10	1/3	10^9^ ≤ *Ra*_*L*_ ≤ 10^13^
180°	0.52	1/5	10^4^ ≤ *Ra*_*L*_ ≤ 10^9^, *Pr*_*l*_ ≥ 0.7

**Table 2:** Natural-convection correlation coefficients for the model parameters and values used in the numerical calculations.

Symbol	Description	Value	Unit
*System conditions and geometry*			
*g*	Gravitational acceleration	9.81	$m\;s^{-2}$
*p*	System pressure	1.01325 × 10^5^	Pa
*T*_sat_	Saturation temperature of nitrogen	77.36	K
*p*_*c*_	Critical pressure of nitrogen	3.3978 × 10^6^	Pa
*T*_*c*_	Critical temperature of nitrogen	126.19	K
*L*_*c*_	Minimum characteristic dimension of the Cu surface	4.0 × 10^−3^	m
*θ*	Surface orientation	0,90,180	°
*Copper-surface properties*			
*k*_Cu_	Thermal conductivity of copper	450	$W\;m^{-1}\;K^{-1}$
*R*_*a*_	Arithmetic mean surface roughness	0.07	μm
*Saturated liquid-nitrogen properties*			
$\rho_{l},\;\rho_{f}$	Bulk-liquid density and saturated-liquid density	806.1	$\mathrm{kg}\;m^{-3}$
$\mu_{l},\;\mu_{f}$	Dynamic viscosity of the liquid	0.161 × 10^−3^	Pas
$c_{p,l},\;c_{p,f}$	Specific heat of the liquid	2042	$J\;{\mathrm{kg}}^{-1}\;K^{-1}$
$k_{l},\;k_{f}$	Thermal conductivity of the liquid	0.145	$W\;m^{-1}\;K^{-1}$
*σ*	Surface tension	8.85 × 10^−3^	$N\;m^{-1}$
*h*_*fg*_	Latent heat of vaporization	199.2 × 10^3^	$J\;{\mathrm{kg}}^{-1}$
*Saturated nitrogen-vapor properties*			
*ρ*_*g*_	Vapor density	4.6121	$\mathrm{kg}\;m^{-3}$
*μ*_*g*_	Dynamic viscosity of the vapor	5.444 × 10^−6^	Pas
*c*_*p*,*g*_	Specific heat of the vapor	1040	$J\;{\mathrm{kg}}^{-1}\;K^{-1}$
*k*_*g*_	Thermal conductivity of the vapor	7.1876 × 10^−3^	$W\;m^{-1}\;K^{-1}$
*Universal constant*			
*σ*_SB_	Stefan–Boltzmann constant	5.67 × 10^−8^	$W\;m^{-2}\;K^{-4}$
*Transformation parameters*			
*η*_1_	CHF superheat scaling factor	$1.05\;\left(S_{1}\right),\;1.10\;\left(S_{2}\right)$	–
*η*_2_	CHF heat-flux scaling factor	$1.50\;\left(S_{1}\right),\;1.80\;\left(S_{2}\right)$	–
*ξ*_1_	MHF superheat scaling factor	$1.10\;\left(S_{1}\right),\;1.20\;\left(S_{2}\right)$	–
*ξ*_2_	MHF heat-flux scaling factor	$1.10\;\left(S_{1}\right),\;1.20\;\left(S_{2}\right)$	–

## Data Availability

The original contributions presented in this study are included in the article. Further inquiries can be directed to the corresponding author.

## Abbreviations

The following abbreviations are used in this manuscript:

AAO	Anodic aluminum oxide
BP	Buckypaper
CHF	Critical heat flux
CNT	Carbon nanotube
FB	Film boiling
HTS	High-temperature superconducting
MHF	Minimum heat flux
MWCNT	Multi-walled carbon nanotube
NB	Nucleate boiling
NC	Natural convection
ONB	Onset of nucleate boiling
PEI	Polyethyleneimine
REBCO	Rare-earth barium copper oxide
SS316	Type 316 stainless steel
TB	Transition boiling
